# Cerebrospinal fluid circulating tumor DNA depicts profiling of brain metastasis in NSCLC

**DOI:** 10.1002/1878-0261.13357

**Published:** 2022-12-29

**Authors:** Jun Wu, Zhiqiang Liu, Tianxiang Huang, Ying Wang, Meng Meng Song, Tao Song, Gretchen Long, Xiaobing Zhang, Xi Li, Longbo Zhang

**Affiliations:** ^1^ Department of Neurosurgery Xiangya Hospital, Central South University Changsha China; ^2^ National Clinical Research Center for Geriatric Disorders Xiangya Hospital, Central South University Changsha China; ^3^ Department of Neuroscience Erasmus Medical Center, Erasmus University Rotterdam The Netherlands; ^4^ Department of Neurosurgery Yale School of Medicine New Haven CT USA; ^5^ Department of Cellular & Molecular Physiology Yale School of Medicine New Haven CT USA; ^6^ Geneplus‐Beijing Institute China; ^7^ Department of Psychology Florida State University Tallahassee FL USA; ^8^ Hunan Key Laboratory of Pharmacogenetics Xiangya Hospital, Central South University Changsha China

**Keywords:** brain metastasis, cerebrospinal fluid, circulating tumor DNA, liquid biopsy, non‐small‐cell lung cancer

## Abstract

Brain metastasis (BM) genetically diverges from the primary tumor in non‐small‐cell lung cancer (NSCLC). Hence, accurately capturing clinically relevant alterations is pivotal for the delivery of targeted therapies. Circulating tumor DNA (ctDNA) sequencing has emerged as a promising liquid biopsy in the biomarker‐based clinical management of recurrent and extracranial metastatic NSCLC. However, the absence of simultaneous sequencing data from brain metastatic sites prevents the definitive evaluation of the efficacy of ctDNA in representing genetic profiles in BM. Here, we performed parallel genomic comparisons between matched BM and primary tumor DNA, plasma ctDNA, and cerebrospinal fluid (CSF) ctDNA. The results indicated that CSF ctDNA had a greater ability than plasma ctDNA to comprehensively represent the mutational landscape of BM, with CSF ctDNA detecting all BM mutations in 83.33% of patients, while plasma ctDNA was only 27.78%. Mutant allele frequency (MAF) in CSF ctDNA was highly correlated with the tumor size of BM (*r* = 0.95), and the mean MAF in CSF ctDNA was higher than that in plasma ctDNA (38.05% vs. 4.57%, respectively). MAF and tumor mutational burden in CSF ctDNA were strongly associated with those in BM (*r* = 0.96 and 0.97, respectively). Of note, CSF ctDNA had significantly higher concordance with BM than plasma ctDNA (99.33% vs. 67.44%), facilitating the identification of clinically relevant mutations. Moreover, we found that plasma ctDNA has stronger profiling performance, with a concordance of 93.01% in multiple brain metastases, equivalent to CSF ctDNA. Collectively, our study indicates that CSF ctDNA is superior to plasma ctDNA in accurately representing the profiling of single BM. Plasma ctDNA could be an alternative liquid biopsy material to be applied in multiple brain metastatic NSCLC.

## Graphical abstract

Parallel genomic comparisons between matched non‐small‐cell lung cancer (NSCLC) and brain metastasis (BM), plasma circulating tumor DNA (ctDNA), and cerebrospinal fluid (CSF) ctDNA. Our study indicates that CSF ctDNA is superior to plasma ctDNA in accurately representing the profiling of single BM. Plasma ctDNA could be an alternative liquid biopsy material to be applied in multiple brain metastatic NSCLC.

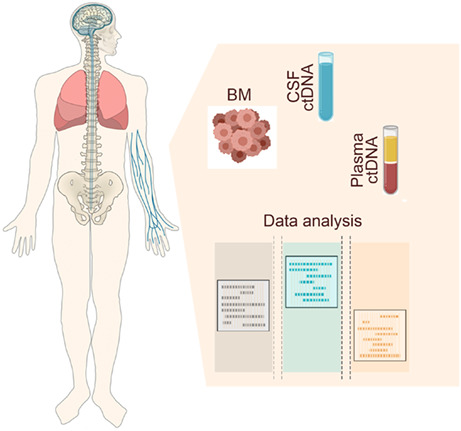

**Abbreviations**
BBB: blood–brain barrier
BM: brain metastasis
CCF: cancer cell fraction
CDKIs: CDK inhibitors
cfDNA: circulating cell‐free DNA
CNS: central nervous system
CSF: cerebrospinal fluid
ctDNA: circulating tumor DNA
LUAD: lung adenocarcinoma
MAF: mutant allele frequency
MDS: molecular residual disease
MRI: magnetic resonance imaging
NSCLC: non‐small‐cell lung cancer
PBLs: peripheral blood lymphocytes
TMB: tumor mutational burden

## 1 Introduction

Lung cancer is the leading cause of cancer‐related mortality worldwide. Non‐small‐cell lung cancer (NSCLC) constitutes 85% of cases of lung cancer, and up to 50% of NSCLC patients will eventually develop brain metastasis (BM) [1, 2]. Recently, biomarker‐guided therapies, enabled by the rapid development of genomic technologies, have inspired targeted anticancer treatments that have shown to dramatically improve NSCLC patient outcomes [3, 4, 5, 6, 7, 8]. However, the prognoses of the patients with BM remain poor. Brain metastatic sites are highly divergent from primary tumors and extracranial metastases although with a common ancestor shared [9, 10, 11]. Hence, accurately identifying potential actionable mutations along with the discovery of acquired resistance mechanisms in real time would tremendously benefit clinical management of NSCLC patients with BM. However, given the challenge of tumor accessibility, it is urgent to establish a minimally invasive biopsy for comprehensively depicting gene abnormalities in BM.

Circulating tumor DNA (ctDNA) has emerged as a promising liquid biopsy in the diagnosis, characterization, and management of metastatic and recurrent cancers by an increasing body of clinical evidence across multiple cancer types [12]. During the last decade, extensive efforts involving the measurement and analysis of ctDNA have promoted its transition from laboratory investigation to clinical application [13, 14, 15]. In lung cancer management, plasma‐derived ctDNA provides a minimally invasive sampling method for real‐time assessment and rapid evaluation at the time of each clinical decision. Beyond identifying therapeutically actionable tumor alterations, dynamic monitoring of ctDNA burden during surveillance serves as an early predictor of response or nonresponse to therapies [12, 14, 16, 17, 18, 19, 20, 21, 22]. Furthermore, ctDNA offers greater potential than conventional biopsy to timely characterize acquired drug resistance since it does not require surgical resection, and enables dynamic measurement and early detection ahead of clinical or radiographic metastasis or relapse [23].

Although ctDNA is a rising star for guiding clinical management in patients with malignancies, some uncertainties remain. The performance of liquid biopsies versus that of simultaneous tumor biopsies has not been studied extensively [12]. Few such studies so far have indicated that plasma ctDNA measurement generally fails to detect ~ 20% of alterations present in the tumor and presumably has an even greater failure in brain tumors [24, 25, 26]. The blood–brain barrier (BBB) is a major anatomical barrier that may limit the performance of ctDNA detection in plasma since it likely prevents tumor ctDNA in the central nervous system (CNS) from reaching the peripheral circulation system. Recent findings also show that high BBB permeability increases the levels of plasma ctDNA [27]. Given the direct contact with the brain and relative ease of access, cerebrospinal fluid (CSF) could have major advantages over plasma for evaluating ctDNA for patients with primary and metastatic brain tumors. Of great clinical significance, several studies have demonstrated that analyzing CSF ctDNA offers a more sensitive and specific source of tumor‐specific mutations compared to plasma ctDNA in primary brain tumors [10, 28]. Furthermore, CSF ctDNA has been recently suggested to be a potentially better option than plasma ctDNA for predicting genomic alterations in leptomeningeal metastasis in NSCLC patients by comparing primary lung cancer, CSF, and plasma ctDNA [29, 30]. However, these pioneering studies are limited by the absence of sequencing data from metastatic sites which prevents the definitive evaluation. To fully explore the efficacy of CSF ctDNA representing genetic profiles in BM versus that of plasma ctDNA in our study, we compared the matched quartet (primary lung cancer, BM, plasma ctDNA, and CSF ctDNA) from the same brain metastatic NSCLC patients.

Our data showed that CSF ctDNA detected all BM mutations in 83.33% of brain metastatic NSCLC patients, whereas only 27.78% in plasma ctDNA. CSF ctDNA was strongly correlated with BM in mutant allele frequency (MAF, *r* = 0.96) as well as tumor mutational burden (TMB, *r* = 0.97). In addition, the mean MAF in CSF ctDNA was significantly higher than that in plasma ctDNA (38.05% vs. 4.57%), suggesting CSF ctDNA offers greater potential to detect genomic alterations in BM due to the limit of detection of the current ctDNA platform. Importantly, CSF ctDNA had significantly higher concordance with BM compared with plasma ctDNA (99.33% vs. 67.44%). A set of clinically relevant BM mutations were absent in plasma ctDNA and solely detected in CSF ctDNA, including *EGFR*, *KRAS*, *RET*, and *CDK13*. Of clinical interest, we found plasma ctDNA had comparable concordance (93.01%) to that of CSF ctDNA to recapitulate mutations only in the cases of multiple brain metastases. Together, our study indicates that CSF ctDNA has greater sensitivity as a high‐quality diagnostic material over plasma ctDNA to depict the comprehensive genomic alterations in BM, and when managing multiple brain metastatic NSCLC, plasma can serve as an alternative material for ctDNA‐based biopsy.

## 2 Materials and methods

### 2.1 Study design and patients

Patients of newly diagnosed lung adenocarcinoma (LUAD) with BM (Xiangya Hospital, 01/2020–01/2022) were involved in this study. All 20 cases were subjected to CT scans (chest and upper abdomen including adrenal glands), MRI (brain), and bone scintigraphy. Tumor DNA was collected from surgical resections (primary lung cancer and BM) or tissue biopsy (primary lung cancer). Brain tumor removal targeted the lesion responsible for refractory intracranial pressure in multiple metastases. Five spatially distinct regions of tumor were heterogenized for tissue DNA extraction. CSF (5 mL via lumbar puncture) was obtained for circulating cell‐free DNA (cfDNA) collection before brain lesion resection. All the patients received the evaluations of general health conditions and the risk of brain herniation before the CSF extraction. The study was approved by the Ethical Committee in Xiangya Hospital (202201017) and the methodologies conformed to the standards set by the Declaration of Helsinki. The experiments were undertaken with the understanding and written consent of each subject.

### 2.2 Histopathology and Ki67 immunohistochemistry

Histopathological diagnosis: digital images of diagnostic tumor sections from all cases were reviewed by at least two pathologists. Ki67 immunohistochemistry analysis: immunohistochemistry was performed using anti‐Ki67 (#9449; Cell Signaling Technology). The percentage of Ki67+ cells was averaged across three tumor sections for each case.

### 2.3 DNA extraction and quantification

DNA of tumor FFPE and peripheral blood lymphocytes (PBLs) were extracted using the DNeasy Tissue and Blood Kit (Qiagen). CfDNA was isolated from plasma and CSF supernatant using QIAamp Circulating Nucleic Acid Kits (Qiagen). The quality control of extracted cfDNA was done by LabChip nucleic acid analysis (total amount ≥ 1.8 ng). The end‐repair was then performed by adding adapters at both ends of the DNA fragment and introducing Index tag to build the ctDNA library. Somatic mutations were obtained by using PBLs as the control sample to filter out germline mutations. DNA concentration was assessed using a Qubit fluorometer (Invitrogen) and the Qubit dsDNA HS Assay Kit (high sensitivity). The size distribution of ctDNA was assessed using Agilent 2100 Bioanalyzer and DNA HS kit (Agilent Technologies).

### 2.4 Library preparation and sequencing

Sequencing library construction for ctDNA was performed by using the KAPA DNA Library Preparation Kit (Kapa Biosystems). Genomic DNA libraries were constructed following the instruction manual of the Illumina TruSeq DNA Library Preparation Kit (Illumina). Libraries were hybridized to custom‐designed biotinylated oligonucleotide probes (Roche NimbleGen) covering ~ 1.1 Mbp of 1021 genes as previously described [31, 32]. DNA sequencing was carried out with GeneSeq2000 with 100‐bp paired‐end reads.

### 2.5 Data analysis and somatic mutations calling

After collection of the sequencing data, clean reads were obtained by removing the terminal adaptor sequences and filtering out low‐quality and too short sequences. Clean reads were aligned to the reference human genome (hg19) using bwa mem. Somatic single nucleotide variants and small insertions and deletions were identified using mutect and gatk software, respectively, by filtering out germline mutations. The candidate variants were obtained by the following criteria: (a) Germline mutations with < 30% AFs in either PBL DNA or ctDNA, (b) nonsynonymous variants with ≥ 1 reads and VAF ≥ 1% in tissue. Nonsynonymous variants with ≥ 2 reads in both blood and CSF [16], (c) Variants < 1% samples found in single nucleotide polymorphism (SNP) databases (dbsnp, https://www.ncbi.nlm.nih.gov/projects/SNP/; 1000G, https://www.1000genomes.org/; ExAC, http://exac.broadinstitute.org/).

### 2.6 Clonal analysis

pyclone software was used to calculate cancer cell fraction (CCF) and cluster all nonsynonymous somatic mutations into putative clonal clusters based on the Bayesian clustering method. Somatic mutations located in the cluster with maximum CCF were defined as clonal and the rest were subclonal.

### 2.7 Statistical analysis

Statistical analyses and graphic visualization were performed using r (version 3.6.0.) and graphpad prism 8. Statistical significance was determined using Wilcoxon matched‐pairs signed‐rank test, Mann–Whitney *U* test (two‐tailed), Friedman's test with multiple comparisons, Pearson correlation, and Fisher's exact test, with *P* < 0.05 for significance for all comparisons. Data are presented as means ± SEM.

### 2.8 Volumetric analyses

Volumetric analyses of BM and peritumoral edema were determined based on enhanced T1‐weighted MRI scans.

## 3 Results

### 3.1 Patient characteristics

We retrospectively profiled 63 samples (primary lung tumor, brain metastasis, peripheral blood, and CSF) from 20 newly diagnosed, untreated, histologically confirmed brain metastatic LUAD patients. Of the 20 patients, 15 had a single BM, whereas the rest of the five patients developed multiple brain metastases (median: 3, range 2–4) diagnosed radiographically (Fig. S1A,B). In addition, two patients had extracranial metastases (liver and thyroid). Demographic and clinical characteristics of the 20‐patient case series are listed in Table S1. The mean sequencing depth of 20 brain metastases and 13 primary lung tumors were 987.65× and 856.15×, respectively (seven primary lung tumors had low‐abundance DNA extraction through biopsy samples or surgical resections had been done in other hospitals). Target sequencing for ctDNA was successfully performed in 19 (95.00%) plasma samples and 11 (55.00%) CSF samples. With a function sequencing depth filtering, 18 plasma ctDNA (mean depth: 1878.89×) and 6 CSF ctDNA data (mean depth: 1643.50×) were proceeded to further analysis.

### 3.2 Genetic divergence of brain metastases and primary tumors

**Fig. 1:**
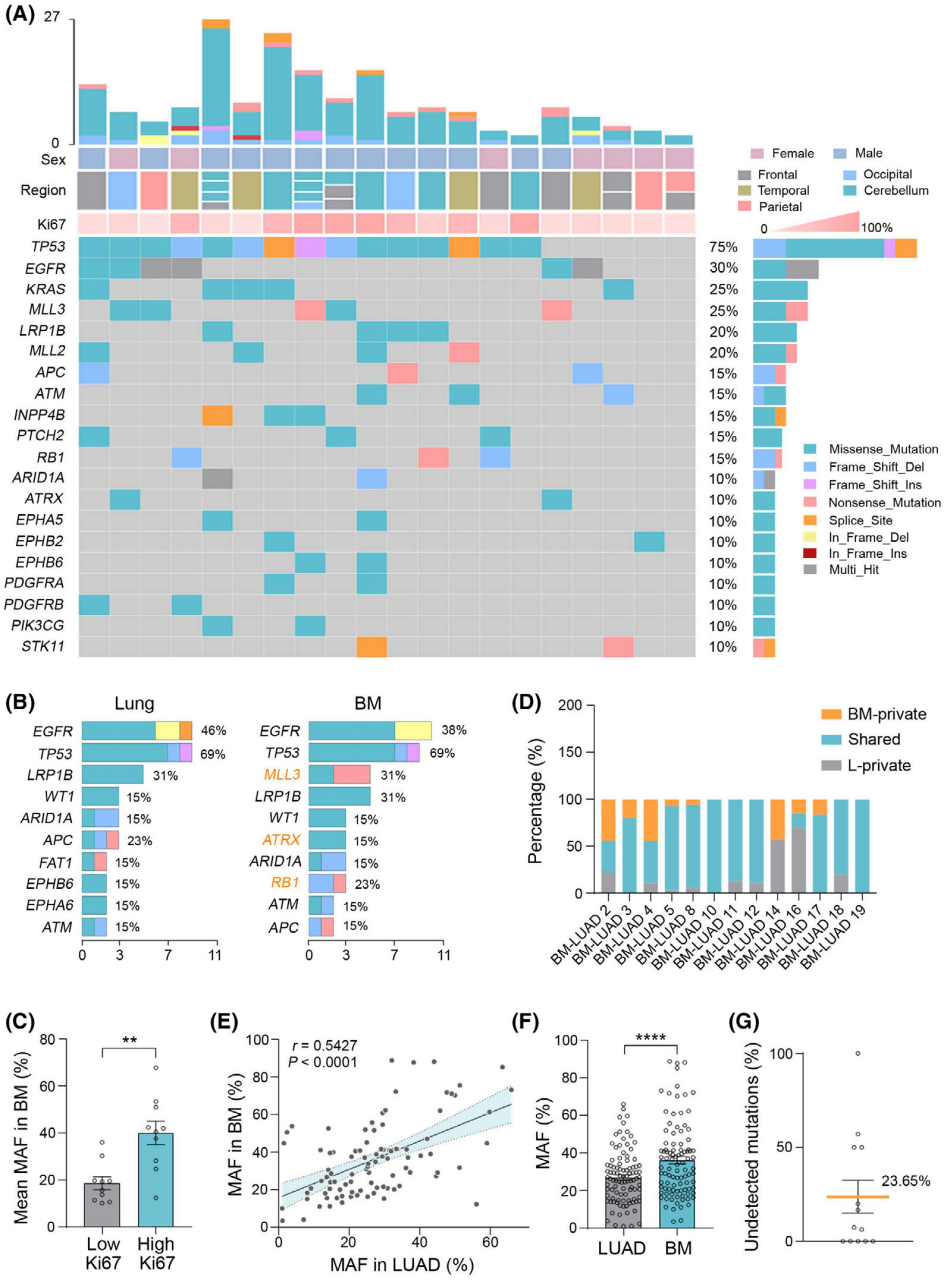
Somatic mutations in primary LUAD and BM. (A) Mutational landscape of brain metastases. TMB is shown in the top bar graph. Top heat maps indicate key patient characteristics including sex, metastatic region and number, and Ki67 index. Bottom heat maps present mutational landscape of top 20 alterations. Mutational rate is depicted in the right bar graph. (B) Beyond high‐frequency mutations shared with the primary tumor, BM has unique frequent mutations in *MLL3*, *ATRX*, and *RB1*. (C) Quantification shows the mean mutant allele fraction (MAF) in BM is significantly higher in lesions with high Ki67 (≥ 30%) expression (Mann–Whitney test, **P ˂ 0.01). (D) Stacked bar graphs depict BM‐private, shared, and LUAD (L)‐private mutations in each individual patient. (E) MAF in BM is correlated with that in primary lung cancer (each circle represents a shared mutation in both BM and primary lung cancer; Pearson *r* = 0.5427, *P* ˂ 0.0001). (F) Bar graph of MAF in primary lung cancer and BM (Wilcoxon signed‐rank test, ****P ˂ 0.0001). (G) Bar graph indicates 23.65% (95% CI: 0–50) mutations in BM were undetectable in primary lung cancer. Data are presented as means + SEM.

Previous studies have demonstrated that BM and their matched primary tumors are clonally related, yet genetically diverse. Each site harbors its own subset of unique mutations [33]. To confirm this divergence in our cohort, we performed a targeted sequencing on primary lung cancer and matched BM. The mean somatic TMB was 9.92 mutations per megabase (Mb) of DNA in primary lung cancer (range: 3–26, median: 5) and 9.35 mutations per Mb in BM (range: 2–27, median: 7.5; Fig. S2A,B). The top 20 mutated genes in BM profiling were highlighted as well as patient characteristics including sex, number, regions of metastatic lesions, and Ki67 index (Fig. 1A, mutational landscape of primary lung cancer shown in Fig. S3). Genetically, BM and primary lung tumor harbored somatic mutations majorly in *TP53* (75.00% BM, 69.23% lung), *EGFR* (30.00% BM, 46.15% lung) and *KRAS* (25.00% BM, 15.38% lung), and BM diverged from primary lung cancer with more frequent alterations in *MLL3*, *ATRX*, and *RB1* (Fig. 1B). Several significant co‐occurring alterations: *EPHA5* (*ARID1A*), *ATM* (*STK11*), *PIK3CG* (*INPP4B*), *ARID1A* (*LRP1B*), and *EPHA5* (*LRP1B*) were identified in BM (Fig. S2C). In addition, our data showed that the mean MAF was positively associated with Ki67 expression in BM, independent of tumor size (Fig. 1C; Fig. S2D). To further evaluate the genetic relevancy and divergence between primary lung cancer and BM, we analyzed the shared and independently evolved mutations in each individual. Results demonstrated that 61.53% (8 of 13) of patients harbored alterations specifically in the BM that were not detected in matched primary cancer (Fig. 1D). The MAFs in BM and primary lung cancer were correlated (Pearson *r* = 0.54, *P* ˂ 0.0001; Fig. 1E), however, the mean MAF in BM was significantly higher than that in primary cancer (36.24% vs. 27.11%, *P* ˂ 0.0001; Fig. 1F). Furthermore, we found that an average of 23.65% (95% confidence interval [CI], 0–50%; Fig. 1G) somatic alterations in BM were not detected in primary lung cancer, including approved actionable mutations such as *EGFR*, molecularly targeted mutations in development such as *ERBB4*, and common tumor suppressor gene abnormalities such as *TP53* and *RB1*. Together, these genetic divergences indicate that profiling primary lung cancer is not sufficient to predict the mutations in BM. However, the accessibility of BM is challenging due to the region and number of brain lesions, the patient's physical status, and the willingness to undergo another invasive sampling. Hence, it is urgent to develop a minimally invasive biopsy procedure to accurately profile BM and identify actionable mutations to assist in targeted drug selection.

### 3.3 Plasma ctDNA is insufficient to recapitulate mutations in brain metastases

**Fig. 2:**
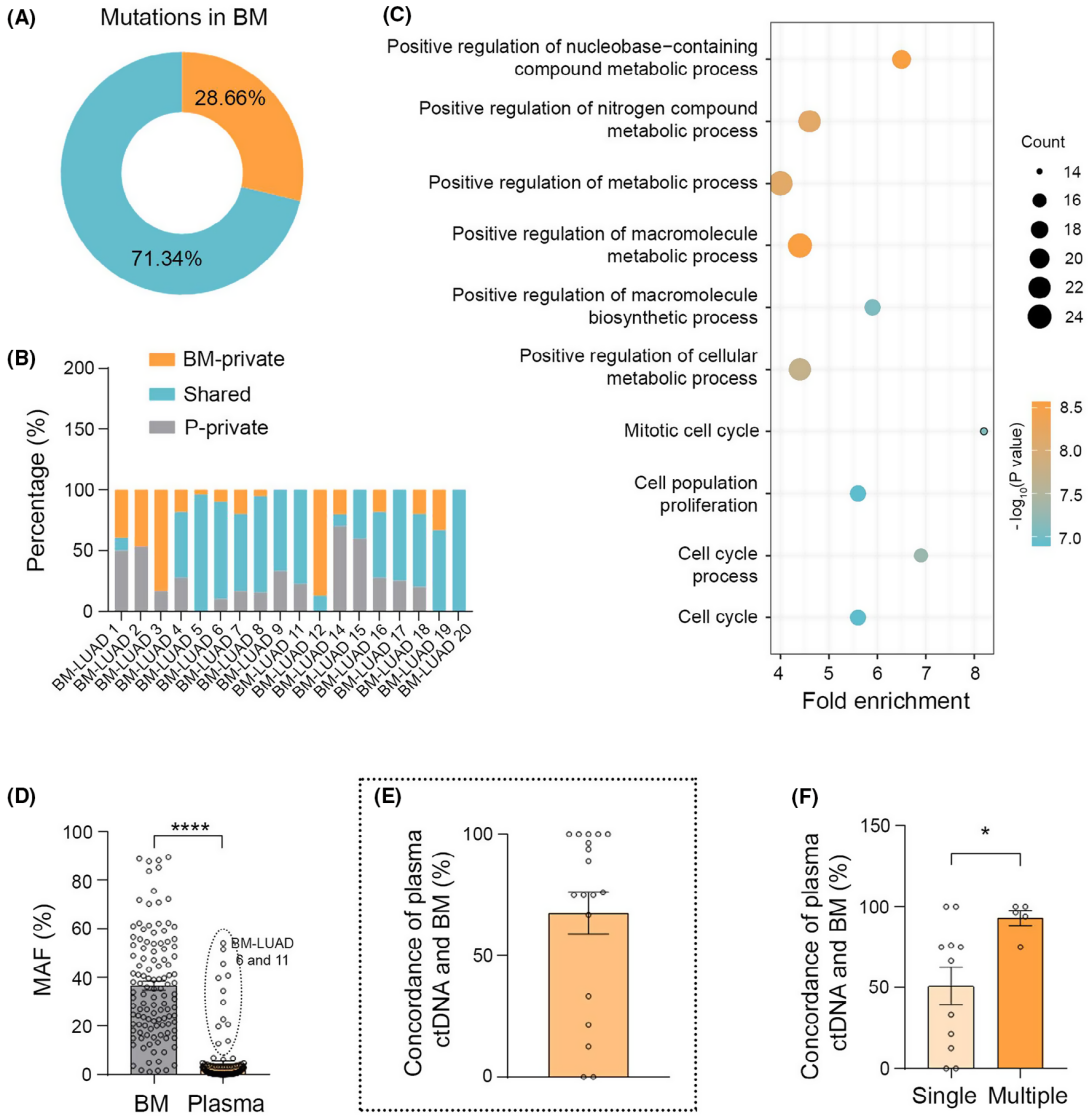
Plasma ctDNA is not sufficient to inclusively describe BM mutations. (A) Pie chart shows the percentage of detected (28.66%) and undetected mutations of BM using plasma ctDNA sequencing. (B) Stacked bar graphs depict BM‐private, shared, and plasma ctDNA (P)‐private mutations in each individual patient. (C) Pathway analysis indicates the undetected genomic alterations are enriched mainly in metabolic and cell cycle‐related processes. (D) Bar graph of MAF of matched mutations in plasma ctDNA and BM. BM‐LUAD 6 and 11 developed extracranial metastases in liver and thyroid, respectively (Wilcoxon signed‐rank test, ****P ˂ 0.0001). (E) Bar graph shows the mean concordance (67.44%) between BM and plasma ctDNA. (F) Quantification indicates that plasma ctDNA has significantly higher concordance with BM in patients with multiple metastases (Mann–Whitney test, *P ˂ 0.05). Data are presented as means + SEM.

Circulating tumor DNA‐based liquid assays from the plasma of peripheral blood samples have demonstrated high concordance of genomic profiles compared to tissue‐based sequencing within and across cancer types [13, 14]. In our study, we detected plasma ctDNA in 92.86% of patients with an average concordance of 64.04% (Fig. S4A,B), which is in line with the previous studies [14, 34, 35]. To test the capability of plasma ctDNA in representing gene alterations in BM, we compared plasma ctDNA with matched BM. The data showed that only 27.78% of plasma ctDNA samples (5 of 18) were successful in detecting all the mutations harbored in matched BM. The unidentified BM mutations by plasma ctDNA was 28.66% (Fig. 2A,B) including actionable gene alterations (41.65% nondetected) such as *EGFR*, *KRAS*, and *RET*, tumor suppressor gene mutations *TP53* and *RB1*, investigational targeted mutations *ERBB4*, *HGF*, and potential biomarker *INPP4B* for guiding immune checkpoint inhibitors [36]. Pathway analysis revealed that the undetected mutations were enriched mainly in metabolic‐related processes, as well as cell cycle processes (Fig. 2C). Although MAF in plasma ctDNA is weakly correlated with that in BM (Pearson *r* = 0.39, *P* ˂ 0.0001), the mean MAF in plasma ctDNA is significantly lower compared with that in BM (4.57% vs. 35.16%, *P* ˂ 0.0001) except in patients with extracranial metastases (BM‐LUAD 6 and 11, liver and thyroid metastases, respectively; Fig. 2D; Fig. S4D,E), which is in line with previous findings where extrathoracic metastasis has a higher MAF detected than no extrathoracic metastasis in NSCLC [24]. We next analyzed the concordance by calculating the percentage of identified BM mutations also found in plasma ctDNA. The data indicated an average of 67.44% (95% CI: 33.33–100) concordance between BM and plasma ctDNA (Fig. 2E). Of note, plasma ctDNA had significantly higher concordance with BM in the patients with intracranial multiple metastatic lesions (93.01% vs. 50.90% in single metastasis, *P* = 0.0449), regardless of sex, TMB, metastatic regions, tumor size, distance to ventricles, or peritumor edema size (Fig. 2F; Fig. S4F,G, Table S2), suggesting the number of intracranial metastatic lesions is the key factor to determine the concordance between BM and plasma ctDNA.

### 3.4 CSF ctDNA analysis is representative of brain metastatic lesion

**Fig. 3:**
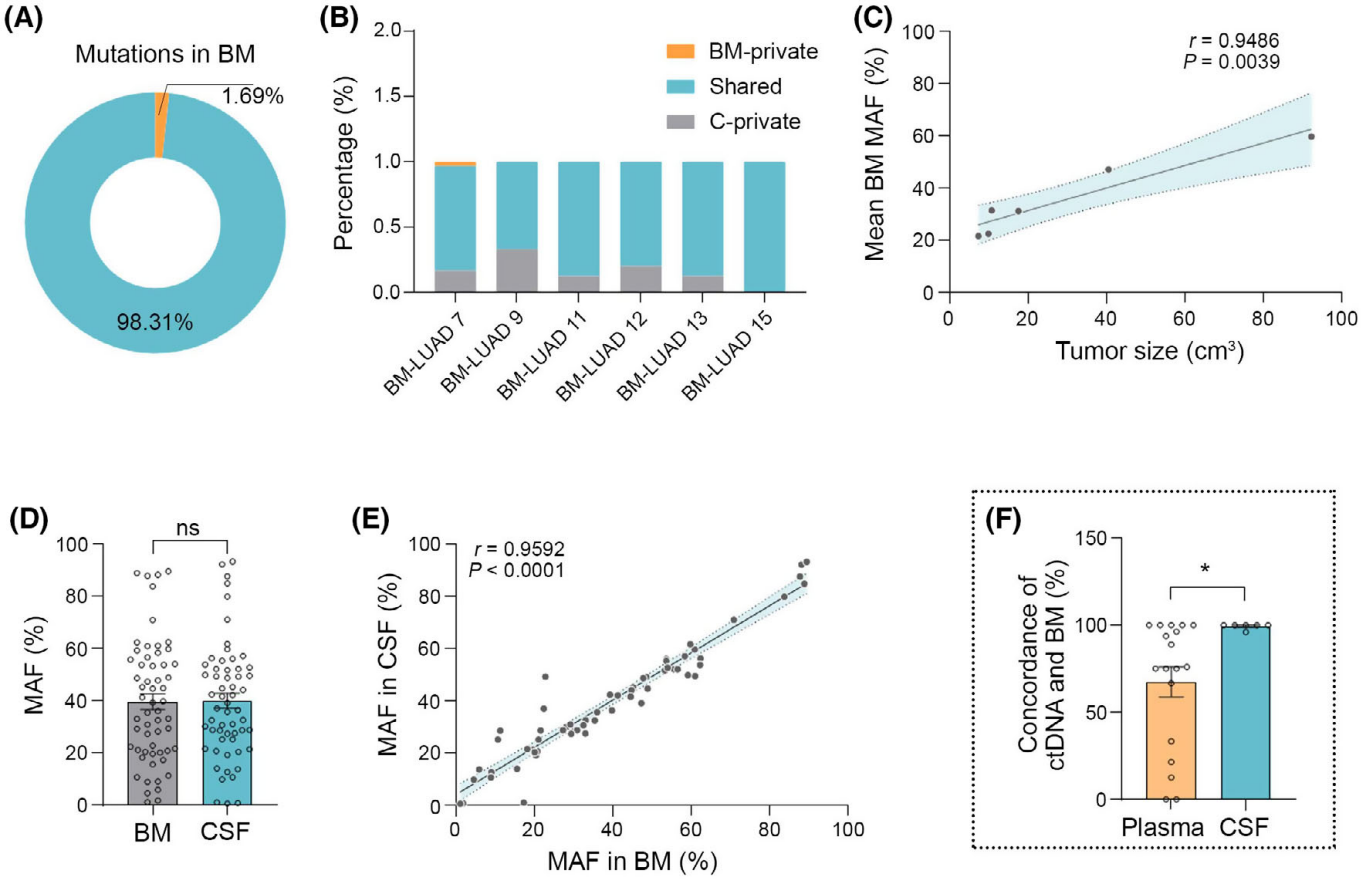
Cerebrospinal fluid ctDNA recapitulates the mutations in single BM. (A) Pie chart indicates that 98.31% of mutations in BM are detectable in CSF ctDNA. (B) Stacked bar graphs depict BM‐private, shared, and CSF ctDNA‐private mutations in each individual patient. (C) Plots show MAF in CSF ctDNA is associated with the size of brain lesion (Pearson *r* = 0.9486, *P* = 0.0039). (D, E) Quantification shows MAF in CSF ctDNA is equivalent and highly associated with that in BM (D: Wilcoxon signed‐rank test, ns: not significant; E: Pearson *r* = 0.9486, *P* = 0.0039). (F) Bar graph of concordance between BM and CSF ctDNA or plasma ctDNA (99.33% vs. 67.44%, concordance data of plasma ctDNA are from Fig. 2E; Mann–Whitney test, *P ˂ 0.05). Data are presented as means + SEM.

Cerebrospinal fluid is a potential alternative to obtain brain tumor ctDNA for analyzing mutations in BM given its direct contact with the brain. CtDNA in CSF is relatively concentrated since the BBB prevents its cycling with the peripheral circulation system [10, 37]. To assess whether the genomic landscape of CSF ctDNA represents mutational profiling in BM, we sequenced the CSF ctDNA obtained from lumbar puncture before brain tumor resection. We found that 83.33% (5 of 6) CSF ctDNA samples recapitulated all the mutations in matched BM (Fig. S5A; 1 of 25 BM alterations was absent in CSF ctDNA sequencing in BM‐LUAD 7). Overall, CSF ctDNA could identify 98.31% mutations harbored in BM with only 1.69% mutations undetected (Fig. 3A,B). The mean MAF in CSF ctDNA is equivalent to that in BM (39.93% vs. 39.58%) and significantly correlated with the size of metastatic lesion by MRI volumetric analyses (Pearson *r* = 0.95, *P* = 0.0039; Fig. 3C,D). In addition, MAF in CSF ctDNA was highly correlated with that in BM (Pearson *r* = 0.96, *P* ˂ 0.0001; Fig. 3E), as well as TMB (Pearson *r* = 0.97, *P* = 0.0003; Fig. S5B,C). To further investigate whether CSF ctDNA profiling is sufficient to represent the alterations in BM, we analyzed the concordance between BM and CSF ctDNA. The result indicated that CSF ctDNA had an average of 99.33% (95% CI: 96–100) concordance with BM, which was significantly greater than that of plasma ctDNA (67.44%, *P* = 0.0125; Fig. 3F). Altogether, these results suggest that CSF ctDNA may prove to be more accurate at profiling BM than plasma ctDNA.

### 3.5 CSF ctDNA has a greater capacity to detect BM mutations than plasma ctDNA for single BM

**Fig. 4:**
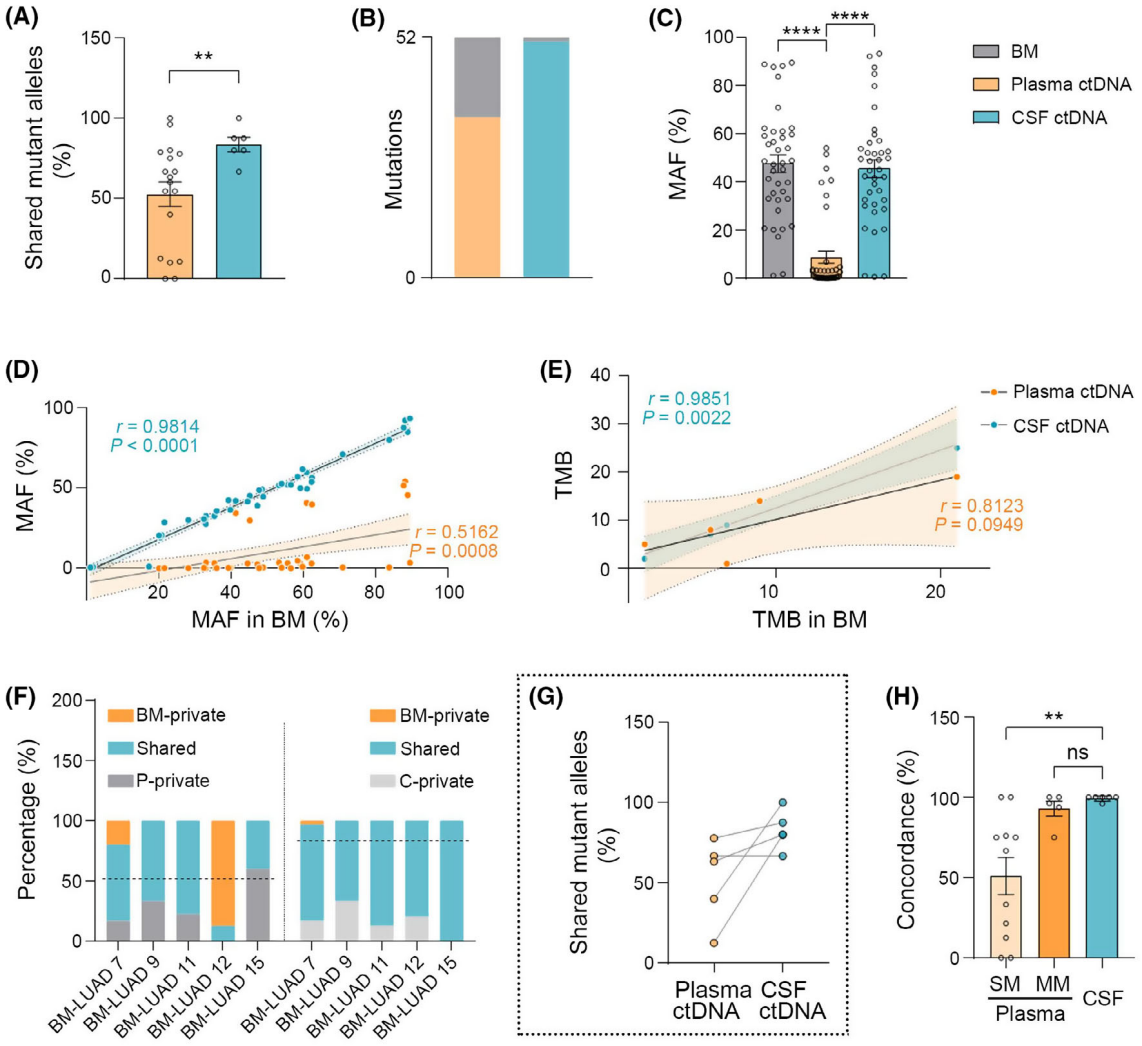
Cerebrospinal fluid ctDNA is better than plasma ctDNA in presenting mutations in single BM. (A) Bar graph of the percentage of shared mutant alleles between BM and plasma ctDNA or CSF ctDNA (percentage was calculated by dividing the number of shared mutations by the total number of mutations detected by sequencing in BM and CSF ctDNA or plasma ctDNA, each circle represents a single patient; Mann–Whitney test, **P ˂ 0.01). (B) Bar graph shows CSF ctDNA detected 51 of 52 alterations in BM, while only 39 alterations were detected in plasma ctDNA (Fisher exact test, *P* = 0.0009). (C) Bar graphs of MAF in matched BM, plasma ctDNA, and CSF ctDNA (Friedman test, ****P ˂ 0.0001). (D) MAF in CSF ctDNA is more strongly correlated with that in BM compared to plasma ctDNA (CSF ctDNA: Pearson *r* = 0.9814, *P* ˂ 0.0001; plasma ctDNA: Pearson *r* = 0.5162, *P* = 0.0008). (E) Plots show TMB in BM has a stronger correlation with that in CSF ctDNA than plasma ctDNA (CSF ctDNA: Pearson *r* = 0.9851, *P* = 0.0022; plasma ctDNA: Pearson *r* = 0.8123, *P* = 0.0949). (F) Stacked bar graphs present BM‐private, shared, and CSF (C) ctDNA‐private or plasma ctDNA (P)‐private mutations in each individual (dotted line: mean). (G) Percentage quantification of shared mutations (with matched plasma ctDNA or CSF ctDNA) in BM (Wilcoxon signed‐rank test, *P =* 0.0625). (H) Bar graphs show that CSF ctDNA and plasma ctDNA have similar concordance with BM in patients with multiple metastases, and significantly higher than that of single metastasis (SM, single metastasis; MM, multiple metastases, Mann–Whitney test; **P ˂ 0.01; ns, not significant). Data are presented as means + SEM.

To further assess whether CSF ctDNA is better than plasma ctDNA in comprehensively representing the mutational landscape of BM, we performed a clonal analysis that showed CSF ctDNA had a higher detection rate of clonal mutations than plasma ctDNA (100% vs. 72.73%; Fig. S6). Comparing the proportion of the shared mutations between BM and plasma ctDNA or CSF ctDNA demonstrated that CSF ctDNA had a significantly higher percentage of shared mutations with BM than plasma ctDNA (mean: 83.61% vs. 54.55%, *P* = 0.0098; Fig. 4A). We then performed parallel analyses for matched trios (BM, plasma ctDNA, and CSF ctDNA) from five patients. The data showed that sampling of brain metastatic lesions detected a total of 52 somatic mutations with 51 identified in CSF ctDNA while only 39 were found in plasma ctDNA (Fisher exact test: *P* = 0.0009; Fig. 4B). CSF ctDNA had equivalent MAF to BM, which was more abundant than plasma ctDNA (Fig. 4C). MAF in CSF ctDNA was highly correlated with that in BM compared with plasma ctDNA (Pearson *r* = 0.98, *P* ˂ 0.0001 vs. 0.52, *P* = 0.0008; Fig. 4D). Moreover, TMB in CSF ctDNA was significantly correlated with that in BM, whereas plasma ctDNA was not (Pearson *r* = 0.99, *P* = 0.0022; Fig. 4E). CSF ctDNA had a higher percentage of shared mutations with BM compared with plasma ctDNA in all five patients (mean indicated by dotted line: 82.83% vs. 52.06%; Fig. 4F,G). Of interest, concordance analyses revealed that plasma ctDNA had a better performance of representing BM alterations in the patients with multiple metastases than those with single metastasis (Fig. 2F). Therefore, we asked whether multiple brain metastases could be an exceptional condition where plasma ctDNA is comparable to CSF ctDNA to depict BM mutations. The concordance result indicated that plasma ctDNA had a similar power as CSF ctDNA to represent alterations in BM in the patients with multiple brain metastases (mean, 93.01% vs. 99.33%; Fig. 4H). Together, these data suggest that CSF ctDNA has an advantage in detecting mutations in BM superior to plasma ctDNA, and plasma ctDNA could be an alternative liquid biopsy material only in multiple brain metastatic NSCLC.

### 3.6 CSF ctDNA facilitates the targeted treatment of brain metastasis

**Fig. 5:**
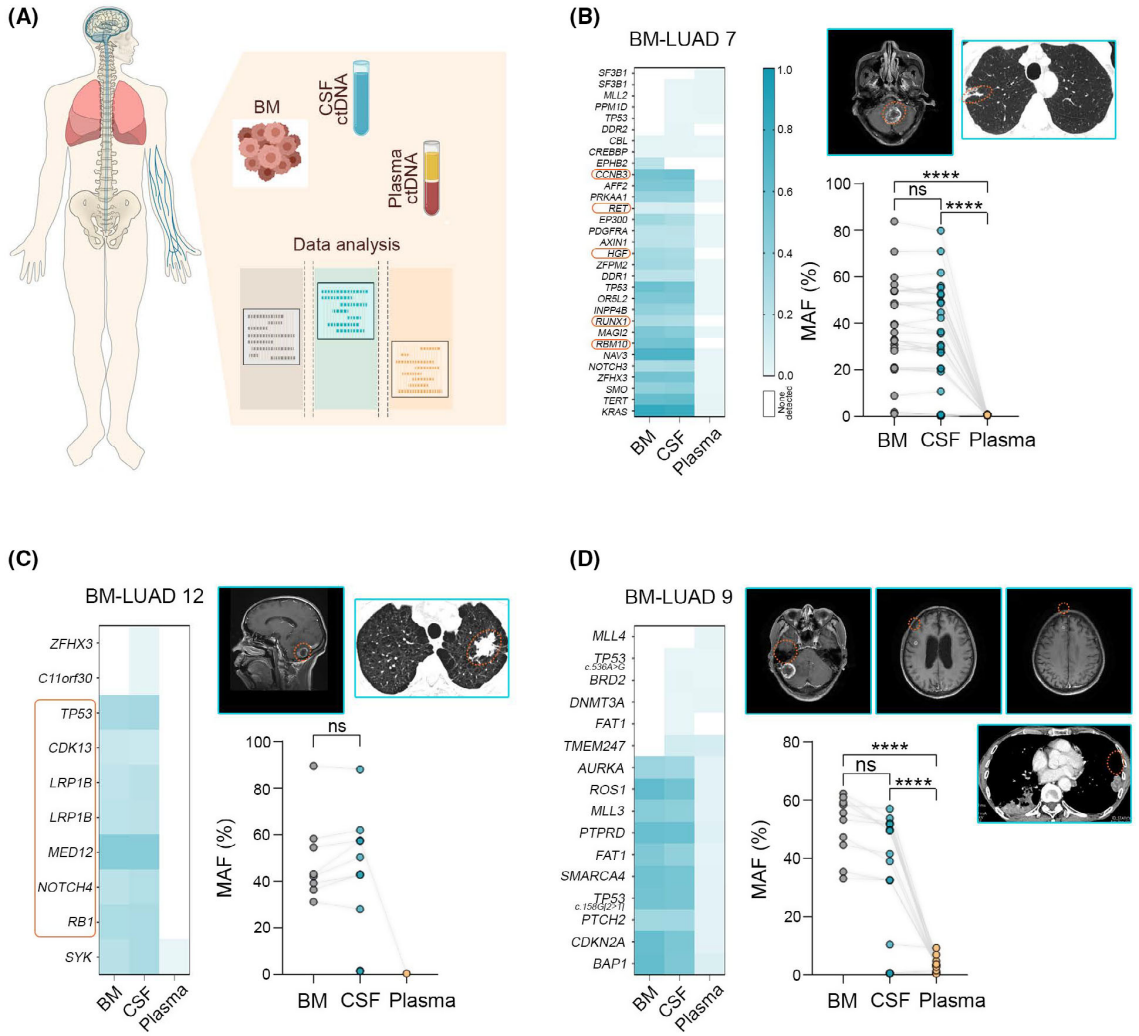
Cerebrospinal fluid ctDNA provides potential molecular targets in advanced lung cancer patients with BM. (A) Diagram of the experimental procedures. (B) Patient BM‐LUAD 7 detected several mutations solely in BM and CSF ctDNA, including actional somatic mutation *RET*. Left: heat maps of detected genomic alterations in BM, CSF ctDNA, and plasma ctDNA; right top: radiographic information (brain: MRI and lung: CT); right bottom: MAF in each sample (Mann–Whitney test, ****P ˂ 0.0001; ns, not significant). (C) Patient BM‐LUAD 12 has mutations in tumor suppressor genes *TP53* and *RB1*, and investigational target genes *CDK13* and *MED12* that were only identified in BM and CSF ctDNA, but not in plasma ctDNA (Mann–Whitney test; ns, not significant). (D) BM‐LUAD 9 confirms that plasma ctDNA is an optional liquid biopsy in the condition of multiple brain metastases in advanced NSCLC (Mann–Whitney test, ****P ˂ 0.0001; ns, not significant).

Identification of actionable mutations in BM is essential for the selection and delivery of targeted therapies to promote favorable outcomes. By comparing profiling results from BM, plasma ctDNA, and CSF ctDNA (diagram: Fig. 5A), we found that CSF ctDNA had a better ability to detect clinically relevant alterations. In BM‐LUAD 7, CSF ctDNA identified an established biomarker, *RET* mutation, which was undetectable in plasma ctDNA (Fig. 5B). In addition, *HGF* alteration was also detected only in BM and CSF ctDNA (Fig. 5B), which is associated with poor prognosis in cancer patients [38, 39]. In BM‐LUAD 12, CSF ctDNA had a greater advantage in detecting a set of somatic alterations that occurred in BM including tumor suppressor gene mutations *TP53* and *RB1*, and investigational targets *CDK13* and *MED12* (Fig. 5C). CDK13 regulates gene transcription and co‐transcriptional processes by phosphorylating the C‐terminal domain of RNA polymerase II, and its selective inhibitor shows promising effects on tumor regression [40]. MED12 is involved in transcription initiation and has been demonstrated to control drug responses in a variety of cancers [41, 42, 43]. Furthermore, patient BM‐LUAD 9 developed three brain metastases located in the frontal lobe and cerebellum. Although MAF in plasma ctDNA here was still significantly lower than that in CSF ctDNA and BM, consistent with our data of plasma ctDNA providing equivalent efficacy to CSF ctDNA in representing profiling of multiple brain metastases (Fig. 4H), all BM mutations were detected in both CSF ctDNA and plasma ctDNA with no significant differences (Fig. 5D). Accordingly, CSF ctDNA is a high‐quality diagnostic material facilitating biomarker‐based therapies for all BM, while plasma ctDNA could be an alternative liquid biopsy only in advanced NSCLC with multiple brain metastases.

## 4 Discussion

Brain metastasis is considered to diverge from primary lung cancer at an early stage. Although both sites share a ‘trunk’ of mutations, the brain microenvironment submits the metastasis to evolve independently to harbor its own subset of unique mutations [33]. In addition, because of poor drug penetration of the BBB, selection pressures can differ and enrich for private subclones within BM that can drive brain tumor progression divergently. Consistent with previous findings [9], our study firstly confirmed that 61.53% of BM harbored clinically relevant mutations that were not identified in the primary tumor, with a mean of 23.65% private mutations nondetected, and many of them associated with sensitivity to targeted therapies either approved or in development such as *EGFR* and *ERBB4* [4, 44]. Brain‐specific evolutionary branching strongly suggested that profiling primary tumor or extracranial metastasis is insufficient to predict the mutational landscape of BM and to guide targeted therapies. Considering the difficulty of brain tumor accessibility, the development and optimization of a minimally invasive biopsy to comprehensively characterize BM for assisting clinical decisions are urgent and crucial.

Advances in ctDNA sequencing and analysis largely lead to the era of precision cancer medicine, with broad applications in identifying therapeutically actionable tumor alterations, sensitive evaluation of early treatment response, dynamic monitoring of tumor recurrence and metastasis, and discovery of mechanisms of acquired resistance. Plasma ctDNA has been validated a number of times as a high‐quality biomaterial of liquid biopsy in detecting and monitoring the mutations of extracranial metastasis in lung cancer. However, the absence of directly profiling the brain tumor limits the definitive comparison between BM and plasma ctDNA. To address the efficacy of plasma ctDNA in representing mutational profiles of brain metastatic sites in NSCLC, we parallelly compared the profiling of BM with plasma ctDNA, and found that more than 70% of patients harbored mutations in BM that were not detectable in plasma ctDNA. The concordance between plasma ctDNA and BM is 67.44% with a set of unidentified alterations including therapeutic biomarkers such as *EGFR*, *KRAS*, and *RET*. In addition, MAF in plasma ctDNA is substantially lower than that in BM, suggesting that tumor DNA in the brain does not circulate well to peripheral blood or is diluted excessively. The BBB is likely the main anatomical contributor preventing both intact tumor cells and tumor cell‐free DNA from reaching the peripheral circulation system. Conversely, the BBB enriches the detection of genomic information inside CSF [45]. We thus explored whether CSF ctDNA is more accurate than plasma ctDNA in detecting BM mutations. Our analyses revealed that MAF in CSF ctDNA was equivalent to that in BM, and more abundant than that in plasma ctDNA, suggesting that CSF has a greater ability than plasma to detect mutations in BM, since low MAF may fall below the physical limits of ctDNA detection. MAF has been demonstrated to be associated with tumor volume [16]. We found MAF in CSF ctDNA, but not plasma ctDNA was highly correlated with the size of brain lesions, which suggests CSF ctDNA can serve as a potential predictor of molecular residual disease (MDS) and treatment response by dynamic measurement. Beyond mutational frequency analysis, our data revealed that TMB in CSF ctDNA was highly correlated with that in BM. TMB has been suggested as a potential predictive biomarker, and its dynamics during treatment can also offer prognostic information for guiding clinical management [46, 47]. Importantly, our parallel analyses indicated an average of 99.33% concordance between BM and CSF ctDNA, which demonstrated the full ability of CSF ctDNA to depict genomic mutations in BM. It is challenging to recapitulate tumor clonal diversity by analyzing a single fragment or even designed multiregion sampling due to the spatial and temporal intratumor heterogeneity [48]. However, ctDNA is considered able to capture tumoral heterogeneity within or across metastatic sites. In our study, CSF ctDNA sequencing identified additional alterations to those detected in BM including *PTEN*, *DDR2*, and *DNMT3A*. *PTEN* mutation is currently an inclusion criterion in five open clinical trials for NSCLC and one clinical trial for malignant CNS neoplasms. *DDR2* mutation serves as an inclusion eligibility criterion in a clinical trial targeting MGCD516, a receptor tyrosine kinase inhibitor, for patients with NSCLC, or metastatic tumors (https://www.mycancergenome.org). Finally, *DNMT3A* is shown to be a potential biomarker associated with the response of immunotherapy and clinical outcome. DNMT3A is shown to be a potential biomarker associated with the response of immunotherapy and clinical outcome [49, 50].

To further investigate the concept of CSF ctDNA in depicting mutational landscape in BM, we analyzed the matched BM, plasma ctDNA, and CSF ctDNA, which demonstrated that MAF in CSF ctDNA was strongly associated with that in BM compared to plasma ctDNA. Meanwhile, TMB in CSF ctDNA, but not plasma ctDNA, was significantly correlated with that in BM. Our data further indicated that CSF ctDNA shared a significantly higher percentage of genomic alterations with BM than with plasma ctDNA. CSF ctDNA detected 51 of 52 detectable mutations in BM, while plasma ctDNA missed 13 mutations, including several actionable mutations with targeted therapies approved or in investigation. In BM‐LUAD 7, *RET* missense mutation was solely detected in CSF ctDNA. RET activation triggers various intracellular signaling pathways such as Ras/RAF and PI3K/AKT, which regulate cell survival, differentiation, proliferation, migration, and chemotaxis [51]. Selpercatinib and pralsetinib are now preferred as the first‐line therapy options for patients with *RET* rearrangement–positive metastatic NSCLC according to NCCN Guidelines [4]. In addition, RET alterations appear to be associated with a high risk of metastasis to the brain [52], and are thought to be exclusive of EGFR, ALK, KRAS, and BRAF mutations, suggesting that it has its own oncogenic driver potential [53, 54]. *HGF* alteration was also detected only in CSF ctDNA. Abnormal activation of HGF could result in dysregulation of HGF/MET signaling. The FDA recently approved MET inhibitor capmatinib as a preferred targeted therapy [4]. In BM‐LUAD 12, we detected common tumor suppressor genes *TP53* and *RB1*, and investigated biomarker *CDK13* and *MED12*. To date, the CDK inhibitors (CDKIs), specifically the ones that block the enzyme activity of CDK12/13 and CDK4/6, have been validated as a promising therapeutic treatment in breast cancer [40, 55, 56]. Accordingly, CSF ctDNA is sufficient and informative to represent the clinically relevant alterations in BM and potentially inform clinical management with superior accuracy to plasma ctDNA. Compared to brain tumor sampling, CSF ctDNA is not anticipated to be limited by sampling frequency, tumor accessibility, or the existence of clinically overt disease. Of clinical interest, by associating with various features, we found that plasma ctDNA has stronger profiling performance in multiple brain metastases, equivalent to CSF ctDNA, suggesting plasma ctDNA could be an alternative liquid biopsy material to be applied in multiple brain metastatic NSCLC.

We acknowledge several limitations to our study. We performed the comparisons and analyses in a relatively small sample volume due to the challenge of BM accessibility. Validations of our findings in larger cohorts are warranted. Consistent with previous reports [57], the CSF ctDNA detection rate here is relatively low (11 of 20 cases before depth filtering; factors associated with the shedding of brain tumor DNA into CSF see ref. [16, 57]). Thus, the improvement of CSF ctDNA collection and sequencing technique would be appreciated. Additionally, the analysis of CSF ctDNA in non‐BM NSCLC patients would be informative on the specificity of the signal origin between plasma and CSF ctDNAs. However, CSF collection in non‐BM NSCLC patients creates a potential ethical issue. Furthermore, our profiling of BM was obtained from accessible lesions during surgical resection in patients with multiple metastases (usually located in the same surgical region). Although spatially and temporally separated brain metastatic sites were suggested to be genetically homogenous [9], future comprehensive profiling of all metastatic lesions would benefit from further evaluation.

## 5 Conclusions

In conclusion, our findings demonstrate that CSF ctDNA is a high‐quality diagnostic material that accurately depicts genomic profiling in BM, with greater sensitivity and specificity than plasma ctDNA, which provides CSF ctDNA a potential to guide clinical strategies of brain metastatic NSCLC management. Plasma ctDNA sequencing might still be an effective alternative for NSCLC patients with multiple brain metastases.

## Conflict of interest

The authors declare no conflict of interests.

## Author contributions

LZ and JW conceived and designed experiments. Surgeries were performed by JW, ZL, TH, YW, and TS; data acquiring and analyzing were performed by LZ, XL, MMS, GL, and XZ; LZ wrote the manuscript; and all authors contributed to reviewing and editing.

## Supporting information


**Fig. S1.** Number and regions of brain metastases.
**Fig. S2.** Integrative analyses of mutations in LUAD and BM.
**Fig. S3.** Mutational landscape of primary lung cancer, brain metastasis, plasma ctDNA, and CSF ctDNA.
**Fig. S4.** Integrative analyses of mutations in plasma ctDNA, lung, and BM.
**Fig. S5.** Tumor mutational burden in BM.
**Fig. S6.** Clonal analysis in matched plasma ctDNA and CSF ctDNA.
**Table S1.** Patient demographic and clinical characteristics.
**Table S2.** Potential factors associated with the concordance between plasma ctDNA and BM.

## Data Availability

Data that support the findings of this study are available from the corresponding authors upon reasonable request.
