# Supplementary material for: Cerebrospinal fluid circulating tumor DNA depicts profiling of brain metastasis in NSCLC
Source: Mol Oncol. 2022 Dec 29;17(5):810–24. doi: 10.1002/1878-0261.13357 (PMC10158766; doi:10.1002/1878-0261.13357)
Supplement: Supplementary file 1 — Fig. S1. Number and regions of brain metastases. Fig. S2. Integrative analyses of mutations in LUAD and BM. Fig. S3. Mutational landscape of primary lung cancer, brain metastasis, plasma ctDNA, and CSF ctDNA. Fig. S4. Integrative analyses of mutations in plasma ctDNA, lung, and BM. Fig. S5. Tumor mutational burden in BM. Fig. S6. Clonal analysis in matched plasma ctDNA and CSF ctDNA. Table S1. Patient demographic and clinical characteristics. Table S2. Potential factors associated with the concordance between plasma ctDNA and BM. [file MOL2-17-810-s001.pdf]

<u>Supplementary Material for:</u> Cerebrospinal fluid circulating tumor DNA depicts profiling of brain metastasis in NSCLC

Jun Wu, Zhiqiang Liu, Tiangxiang Huang, Ying Wang, Mengmeng Song, Tao Song, Gretchen Long, Xiaobing Zhang, Xi Li, Longbo Zhang

Supplementary Material summary:

Suppl. Table S1: Patient demographic and clinical characteristics.

Suppl. Table S2: Potential factors associated to the concordance between plasma ctDNA and BM.

Suppl. Fig. S1: Number and regions of brain metastases.

Suppl. Fig. S2: Integrative analyses of mutations in LUAD and BM.

Suppl. Fig. S3: Mutational landscape of primary lung cancer, brain metastasis, plasma ctDNA and CSF ctDNA.

Suppl. Fig. S4: Integrative analyses of mutations in plasma ctDNA, lung and BM.

Suppl. Fig. S5: Tumor mutational burden in BM.

Suppl. Fig. S6: Clonal analysis in matched plasma ctDNA and CSF ctDNA.

Supplementary Table S1: Patient Demographic and Clinical Characteristics

sex, No. (%)	
Male	13 (65.0%)
Female	7 (35.0%)
Age, years	
Median (range)	61.5 (37-76)
< 60	8 (40.0%)
≥ 60	12 (60.0%)
No. of brain metastases	
1	15 (75.0%)
≥ 2	5 (25.0%)
Regions of brain metastases	
Frontal	11 (33.3%)
Temporal	4 (12.1%)
Parietal	4 (12.1%)
Occipital	3 (9.1%)
Cerebellum	11 (33.3%)
Size of largest brain metastasis (cm^3)	
< 10	12 (60.0%)
≥ 10	8 (40.0%)
Extracranial metastases	
Yes	2 (10.0%)
No	18 (90.0%)

Supplementary Table S2: Potential factors associated to the concordance between plasma ctDNA and BM

Factors	Features	Effect	P value
Sex	Male: 56.25%<br>Female: 43.75%	-0.005	0.984
Ki67 (%)	42.188±30.054	0.363	0.167
Tumor volume (cm^3)	11.610±9.628	0.294	0.269
Distance to ventricles (cm)	1.796±1.037	0.307	0.286
Diameter along ventricles (cm)	3.669±1.722	-0.833	0.421
Peritumoral edema size (cm^3)	105.171±65.994	-0.070	0.812
No. of metastatic lesion	single (68.75%)<br>multiple (31.25%)	0.536	0.032
TMB	8.813±6.911	0.305	0.251
Region	Superior: 62.5%	Reference	
	Inferior: 18.75%	0.095	0.758
	Both: 18.74%	0.488	0.091

Suppl. Fig. 1

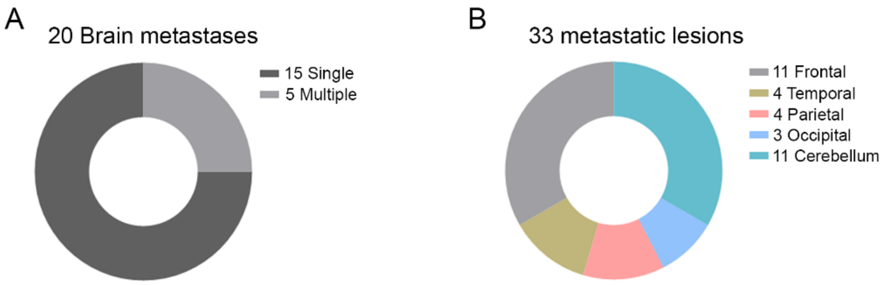

Supplementary figure 1: Number and regions of brain metastases. A: Among the 20-patient case series, 15 cases had single brain metastasis, while the other 5 developed multiple metastases. **B:** Brain regions of the total 33 brain metastatic lesions.

Suppl. Fig. 2

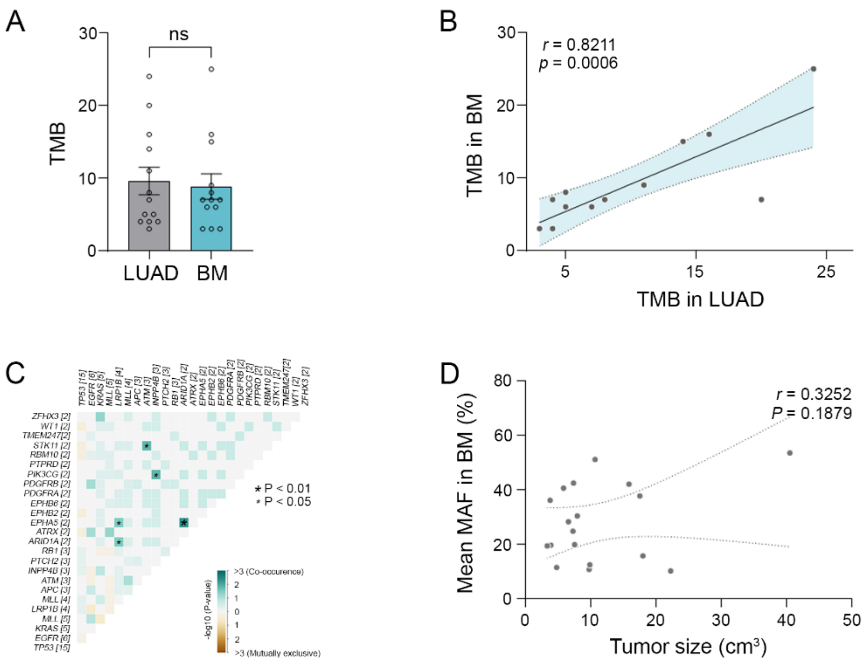

Supplementary figure 2: Integrative analyses of mutations in LUAD and BM. A: Bar graph shows TMB in primary LUAD and BM (Wilcoxon signed-rank test, ns: not significant). **B:** Plots show TMB in LUAD is correlated with that in BM (each circle represents a single patient, Pearson r = 0.8211, P = 0.0006). **C:** Co-occurrence and mutually exclusive alterations in BM (somatic mutation interaction analysis, *: $P < 0.05$, ***:** $P < 0.01$). **D:** Mean MAF in BM is not significantly correlated with brain tumor size (each circle represents a single patient).

Suppl. Fig. 3

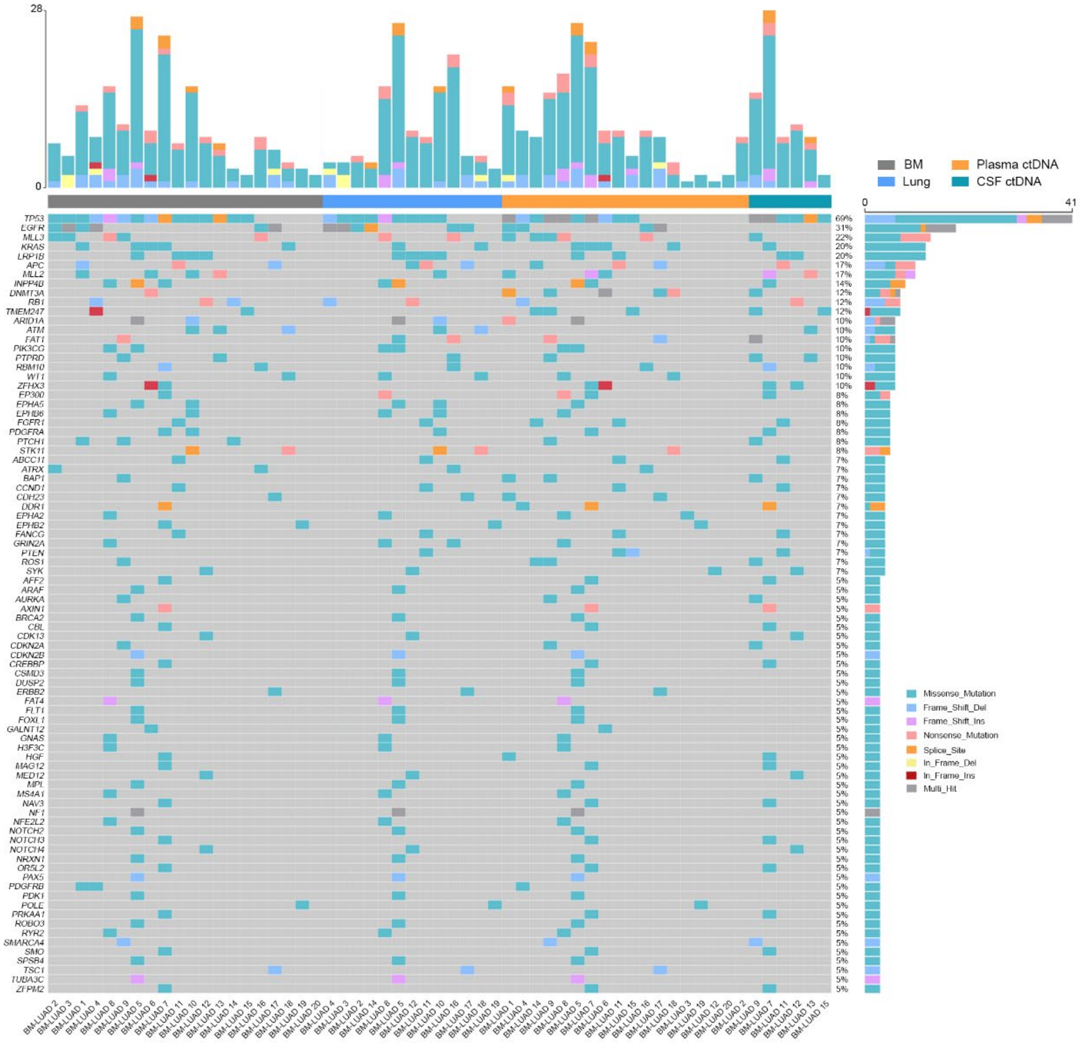

Supplementary figure 3: Mutational landscape of primary lung cancer, brain metastasis, plasma ctDNA and CSF ctDNA. TMB is shown in the top vertical bar graph. Top horizontal bar graph indicates the individual sampling. Bottom heat maps present the mutational landscape. Mutational rate is depicted in the right bar graph.

Suppl. Fig. 4

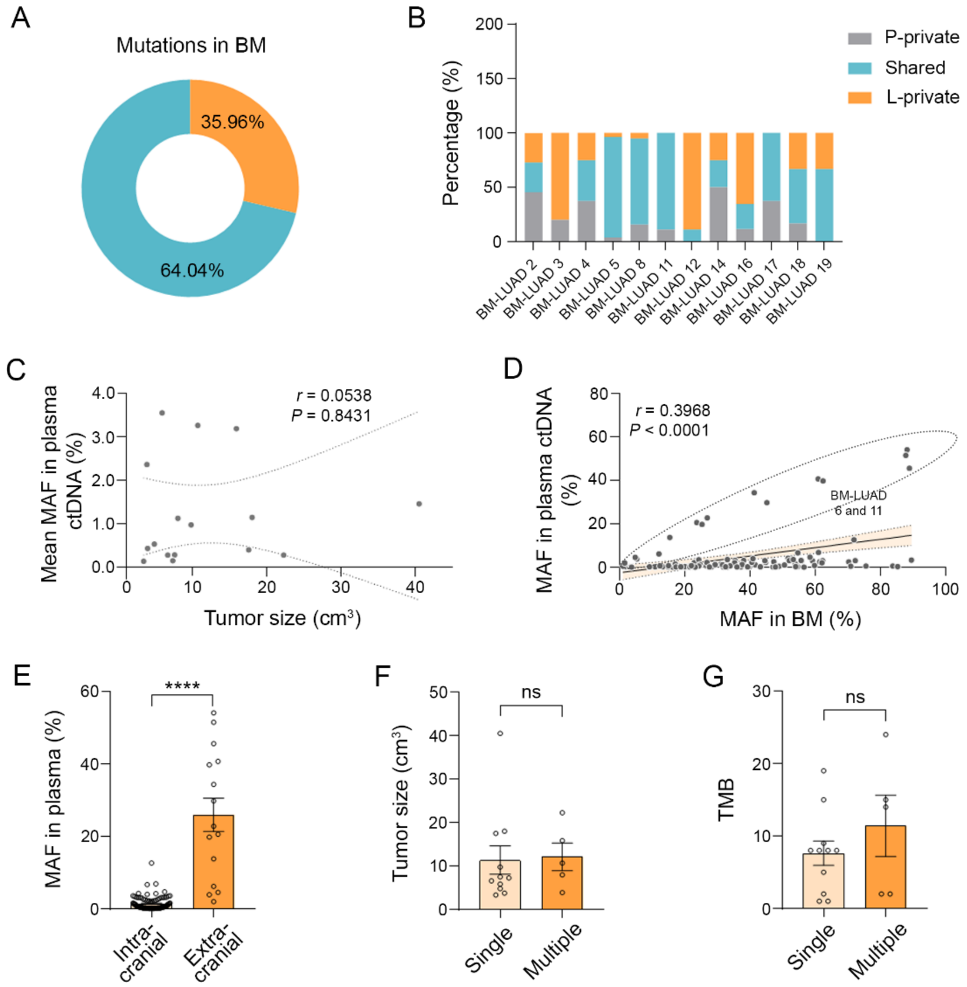

Supplementary figure 4: Integrative analyses of mutations in plasma ctDNA, Lung and BM. **A:** Pie chart shows the percentage of detected (64.04%) and undetected mutations in primary lung cancer using plasma ctDNA sequencing. **B:** Stacked bar graphs depict lung (L)-private, shared and plasma ctDNA (P)-private mutations in each individual patient. **C:** Mean MAF in plasma ctDNA is not correlated with the mass of BM (each circle represents one patient). **D:** Plots show that MAF in plasma ctDNA is associated with that in BM (each circle represents a detected mutation in both BM and plasma ctDNA; Pearson r = 0.3968, P < 0.0001). **E:** Bar graph of MAF in plasma ctDNA in patients with intracranial metastasis or extracranial metastasis. **F:** Bar graph shows that tumor size of a single brain metastasis was not significantly different from that of multiple metastases. **G:** Bar graph of TMB in single brain metastasis and multiple metastases. Mann-Whitney test (C. D. E.); ****: P < 0.0001, ns: not significant.

Suppl. Fig. 5

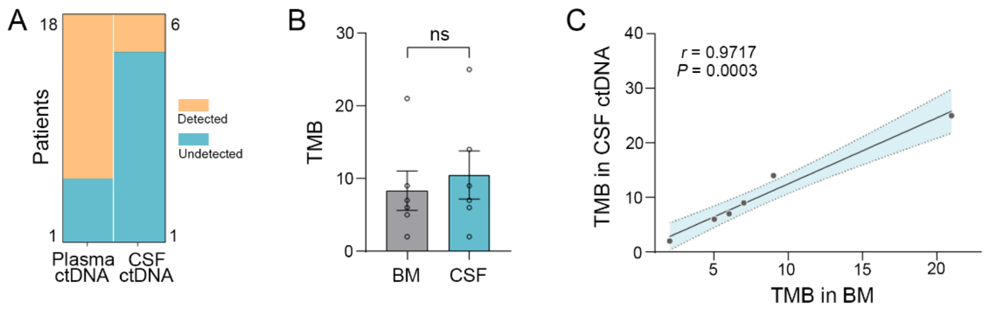

Supplementary figure 5: Tumor mutational burden in BM. A: Heat map of plasma ctDNA and CSF ctDNA in representing all mutations in BM samples (plasma ctDNA: 5 out of 18, 27.78%; CSF ctDNA: 5 out of 6, 83.33%; Fisher's exact test: *P* = 0.0501). **B:** Bar graph of TMB in CSF ctDNA and BM (Wilcoxon signed-rank test, ns: not significant). **C:** Plots show TMB in CSF ctDNA is highly correlated with that in BM (Pearson *r* = 0.9717, *P* = 0.0003).

Suppl. Fig. 6

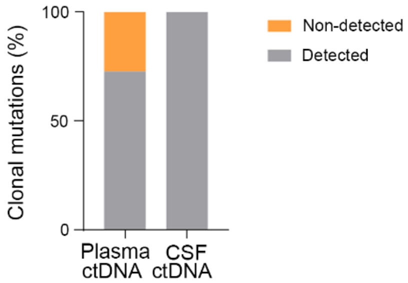

Supplementary figure 6: Clonal analysis in matched plasma ctDNA and CSF ctDNA. Bar graph of the clonal analysis indicates the percentage of BM clonal mutations detected in plasma ctDNA and CSF ctDNA (72.73% vs. 100%).
